# IgG4-related periaortitis presenting as left flank pain

**DOI:** 10.1016/j.radcr.2023.11.003

**Published:** 2023-11-22

**Authors:** Sana Sharrack, Michael Paddock

**Affiliations:** a Department of Rheumatology, Barnsley Hospital NHS Foundation Trust, Barnsley, United Kingdom; b Department of Rheumatology, Sheffield Teaching Hospitals NHS Foundation Trust, Sheffield, United Kingdom; c Medical Imaging Department, Perth Children's Hospital, Perth, Western Australia, Australia; d Division of Pediatrics, University of Western Australia, Perth, Western Australia, Australia; e School of Medicine, The University of Notre Dame Australia, Perth, Western Australia, Australia; f Division of Clinical Medicine, University of Sheffield, Sheffield, United Kingdom

**Keywords:** IgG4-related disease, Autoimmunity, Immunoglobulins, Sialadenitis, Retroperitoneal fibrosis

## Abstract

We present the case of periaortitis which presented initially with left flank pain. A diagnosis of IgG4-related disease (IgG4-RD) was subsequently made and managed as such. IgG4-RD is rare, can be difficult to diagnose, and requires clinical, serological, radiological and pathological correlation, particularly given that serum IgG4 levels may be normal. Immunosuppression is the mainstay treatment for this chronic condition alongside regular rheumatology input.

## Case presentation

**Fig. 1:**
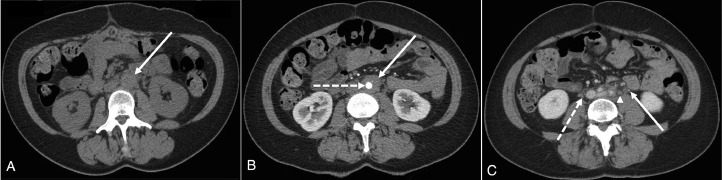
Selected soft tissue window (W:450, L:50) axial computed tomography (CT) slices of a 42-year-old female who presented with left flank pain. (A) Initial noncontrast CT of the kidneys, ureters, and bladder (patient prone) through the level of the kidneys did not demonstrate radiopaque urinary tract calculi. However, subtle low-volume periaortic inflammation (solid white arrow) was identified. The patient was recalled for repeat CT imaging with iodinated contrast. (B) Contrast-enhanced arterial phase imaging at the same level as (A) confirmed circumferential inflammation (solid white arrow) of the contrast opacified aorta (dashed white line) with commensurate narrowing of the aortic caliber from the level of the infrarenal aorta to the aortic bifurcation. There was no aortic ectasia or aneurysmal dilatation. The caliber of the celiac axis, superior mesenteric, and both renal arteries were normal with no periarteriolar inflammation. (C) Portal venous phase imaging at the level of the proximal iliac arteries (inferior to the aortic bifurcation) demonstrated periaortic inflammation extending around the left ureter (solid white arrow; opacified with contrast) which may have been the cause of the patient's left flank pain. An adjacent left para-iliac subcentimeter lymph node (white arrowhead) was also identified alongside a small amount of inflammatory change around the right ureter (dashed white arrow). The appearances were consistent with an isolated periaortitis. A serum erythrocyte sedimentation rate and rheumatology opinion were advised.

A 42-year-old female presented to the Emergency Department (ED) with several weeks’ history of gradually worsening left flank pain and vomiting on a background of feeling generally unwell for 6 weeks. She was afebrile and denied any change in bowel habit, visible hematuria or other urinary symptoms. Her past medical history included previous cauda equina syndrome secondary to a herniated lumbar disc which still caused intermittent right-sided symptoms. There was no other past medical history of note. Her sister had a diagnosis of systemic lupus erythematosus (SLE) but there was no other autoimmune family history. On examination, there was no abdominal guarding or peritonism but there was lower left costochondral junction tenderness on palpation. Initial investigations revealed microscopic hematuria (+++ blood on urine dipstick). Nephrolithiasis was suspected for which the patient proceeded to computed tomography (CT) of the kidneys, ureters and bladder (KUB). No radiopaque urinary tract calculi were identified but subtle periaortic inflammation (Fig. 1) was suspected which was confirmed on post-contrast abdomen and pelvis CT, consistent with an isolated periaortitis.

The serum erythrocyte sedimentation rate (ESR) was elevated (89 mm/h, normal range 1-15 mm/h). During the subsequent rheumatology review, the patient reported a 12-month history of intermittent submandibular gland swelling which lasted for weeks at a time and could affect one or both sides. She complained of xerostomia but no other symptoms of connective tissue disease. There was fullness in the right submandibular region but no lymphadenopathy. There was no synovitis, sclerodactyly, or evidence of a vasculitic rash.

**Fig. 2:**
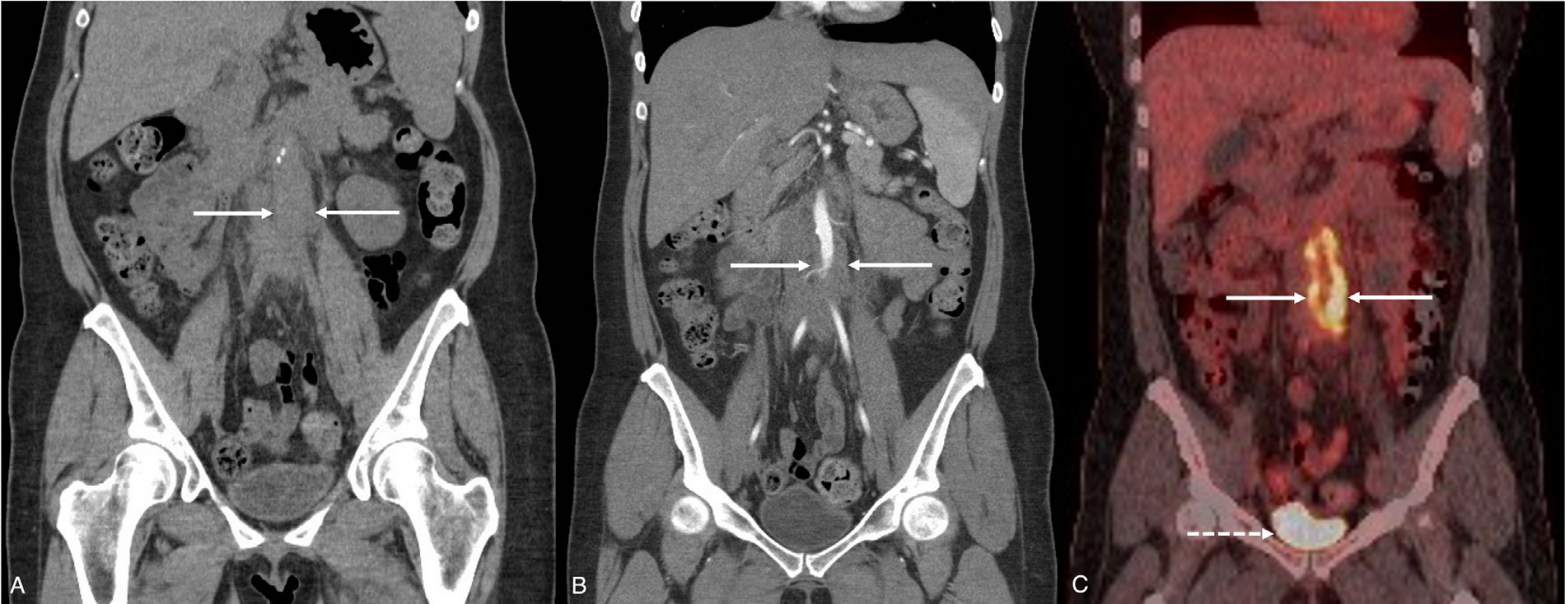
Selected coronal plane computed tomography (CT) imaging through the level of the abdominal aorta in a 42-year-old female who presented with left flank pain. (A) Initial noncontrast CT of the kidneys, ureters and bladder (patient prone) on soft tissue window (W:450, L:50; reconstructed from Fig. 1A) demonstrated subtle periaortic inflammation (between the solid white arrows). Note the foci of intramural calcification in the right infrarenal abdominal aortic wall. (B) Contrast-enhanced arterial phase imaging on soft tissue window (W:450, L:50; reconstructed from Fig. 1B) demonstrated the extent of the circumferential periaortic inflammation from the level of the infrarenal aorta to the aortic bifurcation. The opacified abdominal aorta demonstrated a narrowed caliber secondary to the periaortic inflammation (between the solid white arrows). (C) Corresponding positron emission tomography-computed tomography (PET-CT) acquisition, performed 4 days after (A) and (B), demonstrated intense fluorodeoxyglucose (FDG) activity within the circumferential wall thickening of the abdominal aorta (between the solid white arrows) and the adjacent periaortic fat stranding. Intense activity was demonstrated in the urinary bladder in keeping with expected renal excretion (dashed white arrow). No significant activity was identified elsewhere within the abdomen, pelvis, or other large vessel wall. The appearances were compatible with an isolated large vessel vasculitis or periaortic IgG4-related disease (IgG4-RD).

Subsequent position emission tomography-computed tomography (PET-CT) demonstrated intense activity (Fig. 2) in the previously identified periaortic inflammation (Fig. 1). Appearances were compatible with an isolated large vessel vasculitis or periaortic IgG4-related disease (IgG4-RD), the latter being suspected given the submandibular sialadenitis.

A full autoimmune screen revealed nonspecific homogenous serum anti-nuclear antibody (ANA) positivity (++). All other results were negative, including the immunoglobulin (specifically, the IgG4 level) and infection screen (including syphilis and HIV testing), rheumatoid factor and anti-Sjörgen syndrome (ASS) autoantibodies. A submandibular gland ultrasound +/- biopsy was requested to assess for histological features characteristic of IgG4-RD as an additional aid to the diagnosis.

## Treatment

Our patient was commenced on systemic steroid treatment (oral prednisolone 40 mg once daily) with gastric and bone protection. The submandibular swelling resolved following the commencement of steroids so biopsy was unable to be performed. A clinical diagnosis of IgG4-RD was favored and made in conjunction with the 2019 American College of Rheumatology/European League Against Rheumatism Classification Criteria for IgG4-Related Disease [1] based on the: history; involvement of a typical organ (the infrarenal abdominal aorta i.e., retroperitoneum); major salivary gland involvement; the inability to biopsy; and the classic radiological findings.

## Outcome and follow-up

Our patient reported an improvement in symptom burden following initiation of steroid treatment shortly after presentation. She was started on mycophenolate the following month as her first steroid-sparing disease-modifying antirheumatic drug (DMARD) with subsequent steroid weaning. Methotrexate, a second steroid-sparing DMARD, was introduced a month later. A repeat CT performed 1-year following the initial presentation demonstrated reduced infrarenal abdominal aortic soft tissue inflammation with no change in disease distribution consistent with immunosuppressive treatment response. The renal arteries and suprarenal abdominal aorta remained unaffected.

## Discussion

The diagnosis of IgG4-RD is often made from the combination of clinical, serological, radiological, and pathological information given that biopsy is not often possible. Importantly, IgG4 levels may be normal in a large percentage of patients [1].

### Pathogenesis

IgG4-RD has only recently been recognized as a unifying disease entity linking many previously considered discrete unrelated organ-specific fibroinflammatory conditions [2]. It is thought of as a chronic systemic inflammatory condition of unknown etiology [3]. Nonetheless, the literature recognizes distinct histopathological features including lymphoplasmacytic infiltration, storiform fibrosis, and obliterative phlebitis (amongst others) [3]. It is understood that IgG4 itself is not pathogenic but rather that T helper cells drive disease. Moreover, it is thought that plasmablasts, or other activated B cells in patients with IgG4-RD specifically bind to certain autoantigens resulting in the clonal expansion of certain CD4+ cytotoxic T lymphocytes in specific tissues. This may then lead to fibrosis and inflammation, and consequently organ damage, through either cytokine secretion or induction of apoptosis [3].

### Epidemiology

The incidence of IgG-RD is poorly understood given its very recent identification as a separate disease entity but also because it is often under- or mis-diagnosed [4]. However, it is generally understood that IgG4-RD tends to affect middle to upper-aged patients with an onset of 50-70 years with an overall male predilection [5].

### Clinical presentation

Fibroinflammatory lesions characteristic of IgG4-RD can occur at almost any anatomical site [1] making clinical presentation extremely varied and unpredictable. Nonetheless, there are certain organs which are more likely to be involved, in particular the infrarenal abdominal aorta (as in the case of our patient).

### Imaging findings

Radiological examination is increasingly recognized as an important tool in the identification and diagnosis of IgG4-RD. In the appropriate clinical context, Identifying periaortitis, especially of the infrarenal aorta, should raise suspicion for IgG4-RD.

### Management

IgG4-RD is treatable. Initiation of treatment early is advocated (sometimes urgently depending on the organ involved) when inflammation has not yet resulted in irreversible fibrosis and/or end-organ damage. If left undiagnosed and untreated, significant morbidity and even death can result. Glucocorticoid treatment is the mainstay of initial treatment given its quick action and high response rate in remission induction [4]. A steroid-sparing agent (such as azathioprine, methotrexate, mycophenolate, 6-mercaptopurine, or cyclophosphamide) is often introduced to the treatment regimen to reduce the risks associated with long-term steroid use and to prevent relapse once steroid treatment has been weaned. Rituximab monotherapy has also produced promising results [4]. Many patients require maintenance therapy in the form of a low-dose glucocorticoid +/- a steroid-sparing agent, or a steroid-sparing agent in addition to maintenance rituximab [4]. In those patients taking long-term glucocorticoids, it is important to address bone health and other risks that accompany excess and/or long-term steroid use.

## Conclusion

IgG4-RD is a rare but recently recognized unifying disease entity. It can be difficult to diagnose, especially when biopsy is not possible. Clinical, serological, radiological, and pathological correlation is required, and a large proportion of patients have normal IgG4 levels. Given that several conditions may present with flank pain or “renal colic,”, in the absence of imaging evidence of urolithiasis, the clinical picture should be reassessed with close liaison between clinical teams and radiologists.

## Author contributions

Guarantor of integrity of the entire study: MP; Study concepts and design: MP; Literature research: SS; Clinical studies: MP; Data analysis: n/a; Statistical analysis: n/a; Manuscript preparation: SS, MP; Manuscript editing: SS, MP.

## Patient consent

I, on behalf of all authors, confirm that written, informed consent for publication of this case was obtained from the patient.

## Data Availability

All data supporting the findings of this study are available within the paper.
